# Machine learning classification of fNIRS signals in patients with major depressive disorder and healthy controls

**DOI:** 10.3389/fpsyt.2026.1767469

**Published:** 2026-09-23

**Authors:** Zhuoyan Shan, Xia Liu, Xinyu Lin, Hao Yu, Shanling Ji

**Affiliations:** 1 School of Mental Health, Jining Medical University, Jining, Shandong, China; 2 Shandong Daizhuang Hospital, Jining, Shandong, China; 3 Sleep Medicine Center of Shandong Daizhuang Hospital, Jining, Shandong, China

**Keywords:** functional connectivity, functional near-infrared spectroscopy, machine learning, major depressive disorder, support vector machine

## Abstract

**Objective:**

To determine whether combining resting-state and the verbal fluency task (VFT) prefrontal fNIRS features with machine learning can distinguish patients with MDD from healthy controls (HCs), and to clarify the relative contributions of functional connectivity (FC) versus amplitude features to classification.

**Methods:**

We enrolled 98 patients with MDD and 51 HCs, matched on age, sex, education, and handedness. Participants completed a resting-state fNIRS recording and a VFT using a multichannel prefrontal–temporal montage. After preprocessing, oxyhemoglobin (HbO)–based amplitude features and Pearson correlation–based FC features were extracted from two conditions. Five classifiers—logistic regression, k-nearest neighbors, support vector machine (SVM), partial least squares, and linear discriminant analysis—were evaluated using nested stratified cross-validation. Model performance was quantified using out-of-fold (OOF) predicted probabilities from the outer loop and summarized by accuracy, sensitivity, specificity, and area under the ROC curve (AUC). The 95% confidence intervals (95% CIs) for AUC were estimated using DeLong’s method. The classification threshold was determined using the Youden index on the OOF ROC curve. SHAP analysis was then applied to the best-performing model to quantify the contribution of each feature to the predicted probability of MDD.

**Results:**

Demographic variables did not differ significantly between groups. Across all classifiers, the SVM achieved the best discrimination between MDD and HCs, with OOF AUC of 0.867 (95% CI: 0.805–0.928) and an accuracy of 0.839 (95% CI: 0.770–0.894). At the Youden-optimal threshold (0.482), the SVM model yielded a sensitivity of 0.918 (95% CI: 0.845–0.964) and a specificity of 0.686 (95% CI: 0.541–0.809). SHAP analysis indicated that the top-ranked predictors were predominantly FC features from both resting-state and VFT conditions, particularly IPFC–TPC connectivity at rest and FPC–TPC and IPFC–DLPFC connectivity during VFT, whereas regional HbO amplitude measures contributed less.

**Conclusion:**

The present study suggests that network-level dysconnectivity, which fNIRS captures, plays a central role in distinguishing MDD from HCs, rather than isolated regional hypoactivation. With its high sensitivity, low cost, and SHAP-based interpretability, the SVM model provides an interpretable framework for investigating fNIRS-based neurophysiological patterns associated with MDD and may serve as a potential adjunctive approach for future screening applications after external validation. Multi-center external validation is required before routine implementation.

## 1 Introduction

Major depressive disorder (MDD) is one of the most prevalent and disabling psychiatric illnesses, affecting approximately 3-5% of the global population and representing a leading cause of worldwide disability (1). Despite advances in clinical assessment, the diagnosis of MDD remains largely dependent on subjective symptom reports and clinician interpretation, and reliable neurobiological biomarkers that could support objective identification or stratification are still absent (2). This gap highlights an urgent need for accessible, physiological markers that can complement traditional clinical evaluation.

Functional near-infrared spectroscopy (fNIRS) has emerged as a practical, portable, and non-invasive neuroimaging technique capable of quantifying cortical hemodynamic responses. By measuring changes in oxyhemoglobin (HbO) and deoxyhemoglobin (HbR), fNIRS provides an indirect index of neural activity, particularly within superficial cortical regions such as the prefrontal cortex (PFC) (3). As the PFC plays a central role in emotion regulation and executive function, fNIRS offers a promising avenue for capturing depression-related neurophysiological alterations in both resting-state and task-evoked conditions (4). For example, during the verbal fluency task (VFT) – a common paradigm used to assess executive function – individuals with MDD show significantly reduced frontal HbO responses, often proportional to symptom severity (5). Beyond its potential for identifying depression-related abnormalities, fNIRS has also demonstrated clinical relevance in monitoring treatment-associated neurophysiological changes. Previous studies have reported that improvements in depressive symptoms following treatment were accompanied by increased prefrontal HbO responses, suggesting that fNIRS signals may reflect dynamic changes associated with clinical recovery (6, 7). These findings further support the potential application of fNIRS not only as a diagnostic tool but also as a biomarker for evaluating treatment response in MDD. Nevertheless, conventional univariate statistical approaches based mainly on regional activation differences have shown limited ability to provide robust classification of MDD (8). These approaches often fail to capture the multidimensional and nonlinear characteristics embedded in fNIRS time series and functional connectivity (FC) patterns (9).

In recent years, machine learning (ML) approaches have shown strong potential for extracting complex neurophysiological signatures from high-dimensional brain data, positioning ML as a promising method to enhance the diagnostic utility of fNIRS (10). However, despite increasing evidence supporting fNIRS-based ML approaches, challenges remain regarding biological interpretability, cross-study generalizability, and clinical translation. By leveraging multivariate patterns across channels, task conditions, and the FC features, ML models can achieve more accurate and robust discrimination between patients with MDD and HCs. Recent studies combining fNIRS with ML have demonstrated that multivariate hemodynamic features can successfully distinguish MDD patients from HCs with accuracies often exceeding 80% (2, 10–12).

However, existing fNIRS-based ML studies vary considerably in acquisition paradigms, feature definitions, and validation strategies. Therefore, the present study aims to integrate resting-state and VFT-derived fNIRS features, including regional HbO amplitude and FC measures, to construct ML models for distinguishing patients with MDD from HCs. Additionally, we apply SHAP analysis to identify the features contributing most strongly to model predictions and facilitate their neurophysiological interpretation.

## 2 Methods

### 2.1 Design and population

This study included 149 participants, consisting of 98 patients with major depressive disorder (MDD; 50 females, 48 males) and 51 healthy controls (HCs; 21 females, 30 males). All participants met the inclusion and exclusion criteria described below. MDD patients were recruited from Shandong Daizhuang Hospital between October 2024 and October 2025. All MDD patients were assessed by at least two experienced psychiatrists or senior psychiatric specialists, based on the diagnostic criteria for depression according to the International Classification of Diseases, 10th Edition (ICD-10). The patient group enrolled participants who met the above diagnostic criteria, had a HAMD-17 score ≥ 17, with at least primary school education, and were right-handed. This group excluded patients with other mental disorders, a history of psychoactive substance abuse or dependence, or severe brain organic diseases.

HCs were recruited through public advertisements and were required to have at least primary school education, normal or corrected-to-normal vision, and no history of psychiatric, neurological, or major medical disorders. All HCs were screened by a psychiatrist to ensure the absence of mental health conditions or substance abuse.

The study protocol was approved by the Ethics Committee of Shandong Daizhuang Hospital (Approval No. 202311HY-1). Written informed consent was obtained from all participants in accordance with the Declaration of Helsinki.

### 2.2 Data collection

#### 2.2.1 General data collection

Demographic information was obtained from all participants. Age, sex, years of education, and handedness were collected through structured interviews conducted by trained research personnel.

#### 2.2.2 fNIRS data collection

The fNIRS recordings were conducted in a quiet, temperature-controlled room to minimize environmental interference. To further ensure data quality, all measurements were obtained between 10:00 and 11:00 a.m. to reduce circadian variability. During the procedure, participants were instructed to remain relaxed and minimize head movements. fNIRS signals were collected using the NirSmartII-3000 system (Danyang Huichuang Medical, China), which comprises 11 light-emitting probes and 8 photodetector probes arranged on the forehead with a source–detector distance of 3 cm. The system emitted near-infrared light at wavelengths of 730 nm and 850 nm (**Figure 1A**). The optode montage was positioned according to the international 10–20 system, with adjustments made according to individual head size and anatomical landmarks to ensure consistent placement across participants. The distribution of the probes covered the prefrontal cortex (**Figure 1B**).

**Figure 1:**
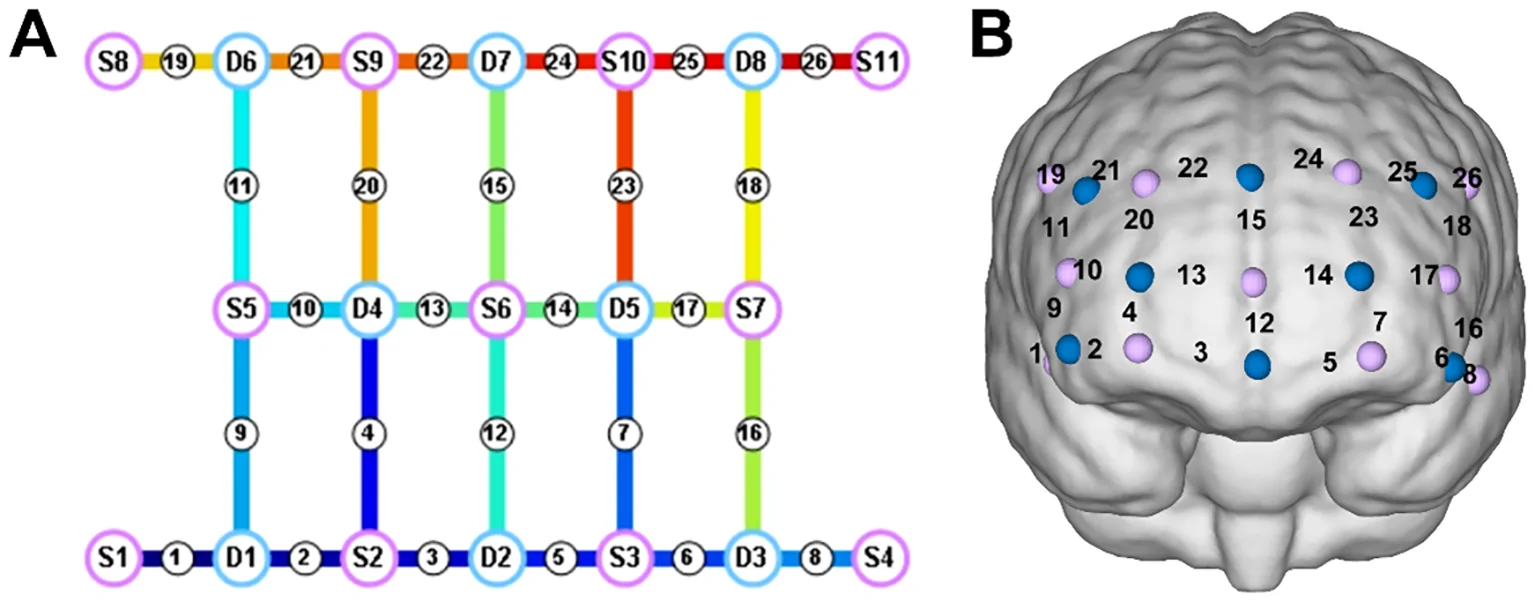
Distribution map of fNIRS emission and reception probes. **(A)** Illustrates the overall distribution of the fNIRS probes, while **(B)** shows their specific placement over the prefrontal cortex. S, Light-emitting probes; D, Receiving probes.

Channels were grouped into five predefined regions of interest (ROIs), including the orbitofrontal cortex (OFC), frontopolar cortex (FPC), temporopolar cortex (TPC), inferior prefrontal cortex (IPFC), and dorsolateral prefrontal cortex (DLPFC), according to the optode montage and previously reported fNIRS channel localization methods (13). Channels 3, 5, and 6 corresponded to the OFC; channels 2, 4, 7, 9, 10, 12, 13, 14, 15, 17, 20, and 23 to the FPC; channel 8 to the TPC; channels 1 and 16 to the IPFC; and channels 11, 18, 19, 21, 22, 24, 25, and 26 to the DLPFC. The sampling rate of the system was 10 Hz.

Before the recording began, participants watched a brief instructional video to familiarize themselves with the experimental procedures and task requirements. Resting-state data were recorded for 10 minutes while participants remained awake and relaxed. The 10-minute resting-state duration was selected based on previous fNIRS resting-state studies, which typically use several-minute recordings to obtain reliable spontaneous hemodynamic fluctuations while maintaining participant comfort (14). For the first 5 minutes, participants closed their eyes; for the subsequent 5 minutes they opened their eyes and fixated on a “plus sign” (+) displayed on a screen. The resting-state scan in fNIRS was designed to capture spontaneous cortical hemodynamic fluctuations in the absence of explicit cognitive tasks, reflecting the brain’s intrinsic functional organization (4).

Participants also performed the Verbal Fluency Task (VFT), which included a 30-second pre-task period for controlling motion artifacts (counting from 1 to 5), a 60-second task period where they generated words starting with a given phonological Chinese characters, and a 70-second post-task period for removing task-related artifacts (counting from 1 to 5). The 60-second task duration was selected based on previous fNIRS-VFT paradigms, as this period is sufficient to induce measurable prefrontal hemodynamic responses associated with language retrieval and executive control processes (14). Participants performed a Chinese character-based VFT, in which they were instructed to generate as many meaningful phrases as possible based on visually presented Chinese characters (e.g., “白” [white], “日” [sun], and “横” [horizontal]). This paradigm differs from traditional semantic category fluency tasks, which require participants to generate words belonging to a predefined category (e.g., animals or fruits). Instead, the present task mainly involves lexical retrieval, language production, and executive control processes, while also incorporating language-specific characteristics of Chinese characters. To minimize silent intervals, the character set was replaced every 20 seconds. The VFT was designed to assess cognitive flexibility and executive function, as it requires the participants to rapidly generate words and phrases, both of which are often impaired in individuals with MDD (15).

### 2.3 Data preprocessing

Raw fNIRS data first underwent a signal quality assessment, during which channels exhibiting low light intensity, saturation, or clear signal dropout were excluded from further analysis. The following fNIRS data were processed using NirSpark software. To reduce data artifacts and sudden fluctuations, appropriate preprocessing steps were applied. Raw fNIRS signals were preprocessed using NirSpark software (Danyang Huichuang Medical, China). Raw fNIRS data were preprocessed using NirSpark software (v1.8.9; Danyang Huichuang Medical, China). Preprocessing included channel quality assessment, removal of unstable segments, motion artifact correction, FIR low-pass filtering, and conversion of optical density signals into HbO and HbR concentrations using the modified Beer–Lambert law. A FIR low-pass filter with a cutoff frequency of 0.2 Hz was applied to reduce high-frequency physiological noise while preserving task-related hemodynamic fluctuations. The optical density values were then converted into concentrations of HbO and HbR using the modified Beer–Lambert law. Two relative concentrations of HbO and HbR were calculated to assess hemodynamic fluctuations in the prefrontal cortex using near-infrared light at specific wavelengths. Given its superior signal-to-noise ratio and high sensitivity to changes in local blood flow, HbO was selected as the primary measure for analysis, as it provides more reliable indicators of cortical activation. This preprocessing procedure ensures the data are clean and suitable for subsequent analysis, improving the accuracy and reliability of the fNIRS measurements (16).

FC analysis was performed at both global and ROI-specific levels: we calculated Pearson correlation coefficients between HbO time series of predefined ROIs (OFC, FPC, TPC, IPFC, DLPFC) under resting-state and VFT conditions. Pearson correlation coefficients were selected as functional connectivity measures because they provide a straightforward and widely used approach for quantifying temporal synchronization between fNIRS channels. Previous fNIRS studies have commonly applied Pearson correlation-based connectivity analysis to characterize inter-regional functional interactions. Although other approaches, such as nonlinear connectivity metrics or graph-based measures, may provide complementary information, Pearson correlation was selected in the present study due to its interpretability and suitability for the current sample size (4). Resting-state data reflect the brain’s intrinsic functional networks, while task-evoked data are more effective in revealing brain activation patterns under specific cognitive tasks. Therefore, we use both resting-state fNIRS data and task activation data simultaneously, taking full advantage of their complementary insights into brain function (17).

### 2.4 Feature extraction

Two categories of features were extracted from the preprocessed HbO signals: amplitude-based measures and FC. Amplitude-based features included the mean HbO concentration during resting-state eyes-closed and eyes-open conditions, the eyes-open minus eyes-closed contrast, the mean HbO level during the task period, and the task–baseline difference computed using the first 20 seconds of the pre-task interval. These measures were designed to capture both spontaneous fluctuations and task-evoked hemodynamic responses.

FC features were computed at both global and ROI levels. Pairwise Pearson correlation coefficients were calculated between HbO time series from the predefined ROIs (OFC, FPC, TPC, IPFC, DLPFC) under both resting-state and task conditions, yielding ROI-to-ROI connectivity matrices that characterize interregional synchronization patterns. To take advantage of the complementary information provided by these two states, both resting-state and task-derived features along with demographic information were used as inputs for the ML models.

### 2.5 Machine learning classifiers and training

Five widely used ML classifiers were evaluated, including logistic regression, k-nearest neighbors (KNN), support vector machine (SVM), partial least squares (PLS), and linear discriminant analysis (LDA). These established ML algorithms were selected because they are suitable for moderate-sized neuroimaging datasets and allow comparison within a transparent and consistent analytical framework. The binary classification outcome was defined as MDD versus HCs, with MDD coded as the positive class.

To obtain a conservative estimate of generalization performance while tuning hyperparameters, we used nested stratified cross-validation. This approach separates hyperparameter optimization from model evaluation and helps reduce selection-related optimism in moderate-sized neuroimaging datasets. Specifically, an outer 10-fold stratified cross-validation loop was used purely for model evaluation. Within each outer training fold, an inner 5-fold stratified cross-validation was performed to select model hyperparameters by maximizing the AUC. Stratification was applied in both the inner and outer loops to maintain approximately consistent MDD-to-HC proportions across folds, which was particularly relevant given the unequal group sizes. Prior to model fitting, continuous features were standardized using parameters derived from the inner training data only, and then applied to the corresponding validation. All feature computation was performed identically across folds, and feature standardization was conducted within the training data of each cross-validation split to avoid information leakage. All models were implemented in R using the caret package, which provided a unified interface for hyperparameter tuning, resampling, model training, and performance evaluation. Nested stratified cross-validation was configured within caret to ensure that hyperparameter optimization in the inner loop and model assessment in the outer loop were fully separated. Feature standardization and preprocessing steps were performed within each fold using caret’s built-in preprocessing functions to prevent information leakage.

For each outer fold, the optimized model generated predicted probabilities for the held-out outer test set. These out-of-fold (OOF) predictions were concatenated across all folds to compute the overall ROC curve and OOF AUC. The classifiers were evaluated using accuracy (ACC), sensitivity (SEN), specificity (SPE), and the AUC. ACC, SEN, and SPE were calculated using the Youden-optimal threshold derived from the OOF ROC curve rather than a fixed cutoff of 0.5. This threshold-selection procedure considered SEN and SPE jointly when defining the operating point, rather than relying on overall ACC alone under unequal class sizes. The 95% confidence intervals (95% CIs) for AUC were estimated using DeLong’s method. The model with the highest OOF AUC was selected as the final classifier for subsequent interpretability analysis.

### 2.6 SHAP analysis

To improve the interpretability of the SVM classifier, SHAP analysis was applied to quantify the contribution of each feature to the model’s predictions. SHAP values were computed using the final SVM model refitted on the complete dataset after nested cross-validation. This refitted model was used exclusively for interpretation of feature contributions and was not involved in estimating predictive performance. Model performance metrics were derived solely from OOF predictions generated during nested cross-validation. SHAP provides a unified framework for assessing feature influence, allowing us to determine how functional and hemodynamic indicators shift the predicted probability toward either the MDD or HC class.

### 2.7 Statistical methods

Data processing and analysis were performed using R (version 4.4.1) and R Studio. Descriptive statistics were used to summarize the demographic and clinical characteristics of the participants. Continuous variables were expressed as the mean ± standard deviation (SD) for normally distributed data and as the median [P25, P75] for non-normally distributed data. Between-group comparisons of continuous variables were performed using independent-samples *t*-tests for normally distributed measures and the Mann–Whitney U test (Wilcoxon rank-sum test) for non-normally distributed measures. Categorical variables were presented as frequencies and percentages, with group comparisons made using the chi-square test. For analyses involving multiple fNIRS and cognitive variables, false discovery rate correction using the Benjamini–Hochberg method was applied to control for multiple comparisons. All tests were two-tailed, and statistical significance was set at *p* < 0.05.

## 3 Results

### 3.1 Demographic characteristics

As shown in **Table 1**, the two groups did not differ substantially in demographic characteristics. All participants in both groups were right-handed. Statistical comparisons confirmed that none of these differences were significant.

**Table 1:** Demographic information (mean ± SD).

Measurements	MDD (n = 98)	HCs (n = 51)	*t*/*χ2*	*p*
Age(years)	34.15 ± 13.28(18,59)	31.24 ± 11.57(18,60)	t = 1.33	0.186
Genders(F/M)	50/48	21/30	χ2 = 0.54	0.46
Years of Education	10.30 ± 3.79	11.12 ± 3.83	t = −1.082	0.279
Handedness (Right/Left)	98/0	51/0	−	−

Age and years of education were compared using independent-samples *t*-tests. Gender was analyzed using the chi-square test; MDD, major depressive disorder; HCs, healthy controls. F/M, Female/Male.

### 3.2 Machine learning analysis

Among all classifiers, the SVM achieved the best discrimination performance. Based on OOF predictions from nested cross-validation, the SVM yielded an AUC of 0.87 (95% CI: 0.81–0.93), with a SEN of 0.92 (95% CI: 0.85–0.96), SPE of 0.69 (95% CI: 0.54–0.81), and ACC of 0.84 (95% CI: 0.77–0.89) (**Table 2**). The ROC curves of the five classifiers are shown in **Figure 2**.

**Table 2:** Model performance metrics.

Model	SEN (95% CI)	SPE (95% CI)	ACC (95% CI)	AUC (95% CI)
SVM	0.918 (0.845–0.964)	0.686 (0.541–0.809)	0.839 (0.770–0.894)	0.867 (0.805–0.928)
Discriminant Model	0.724 (0.625–0.810)	0.765 (0.625–0.872)	0.738 (0.660–0.807)	0.778 (0.704–0.851)
KNN	0.663 (0.561–0.756)	0.784 (0.647–0.887)	0.705 (0.625–0.777)	0.776 (0.699–0.853)
Logistic Regression	0.694 (0.593–0.783)	0.784 (0.647–0.887)	0.725 (0.646–0.795)	0.753 (0.674–0.831)
PLS Model	0.561 (0.457–0.661)	0.843 (0.714–0.930)	0.658 (0.576–0.733)	0.720 (0.638–0.803)

CI, confidence interval; SEN, Sensitivity; SPE, Specificity; ACC, Accuracy; AUC, Area Under the ROC Curve; KNN, K-Nearest Neighbors; SVM, Support Vector Machine; PLS Model, Partial Least Squares Model.

**Figure 2:**
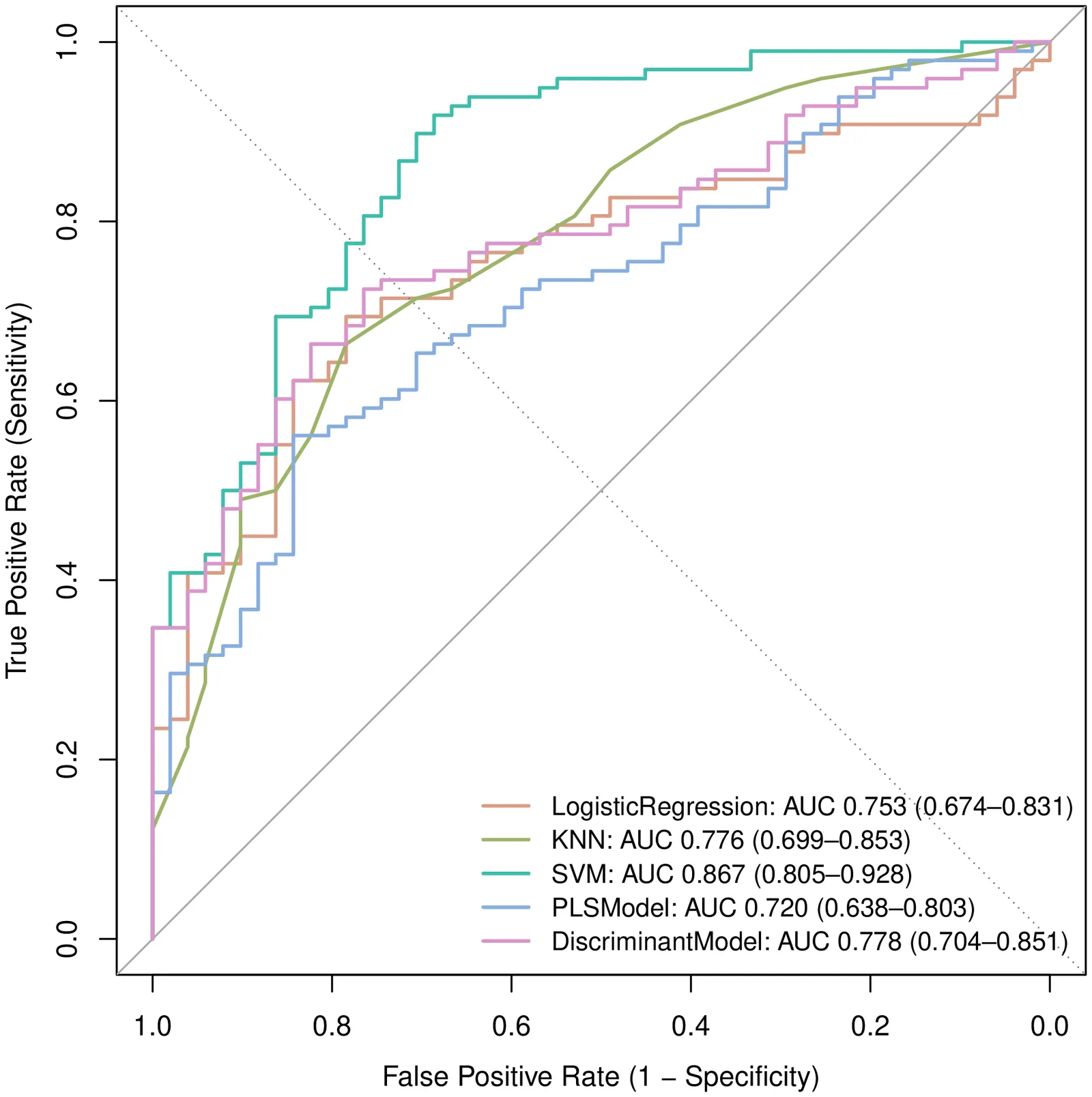
The ROC curve analysis of multiple machine learning models for prediction MDD. KNN, k-nearest neighbors; SVM, support vector machine; PLS, partial least squares.

Overall, the SVM provided the most favorable trade-off between sensitivity and specificity among the evaluated algorithms and was therefore selected for subsequent interpretability analysis.

### 3.3 SHAP analysis for feature importance

The SHAP summary plot (**Figure 3A**) revealed that FC features dominated the model’s decision process. The highest-ranked predictors were FC measures derived from both resting-state and VFT conditions. Among these, IPFC–TPC connectivity during resting state was the strongest contributor, followed by FPC–TPC and IPFC–DLPFC connectivity during the VFT, as well as global FC indices across both paradigms. Age also showed a non-negligible contribution but remained slightly below the top FC features.

**Figure 3:**
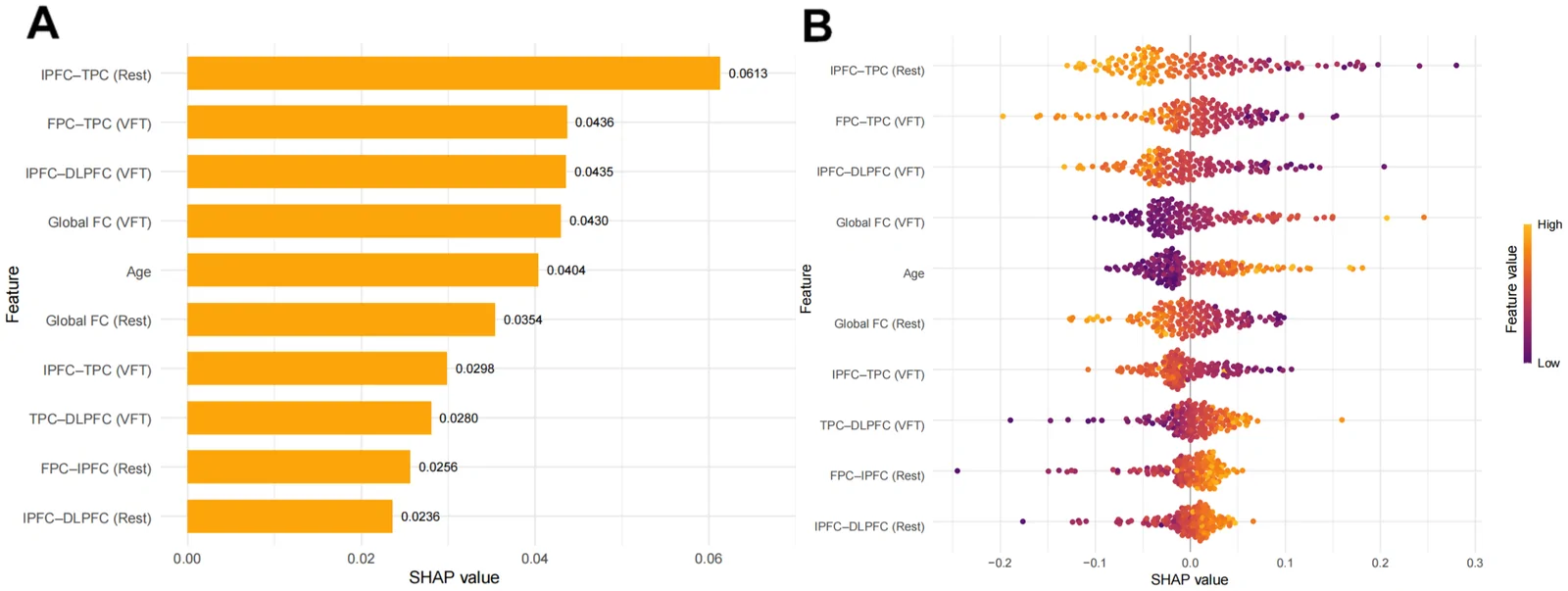
SHAP-based interpretation of the SVM classifier. SHAP summary plot showing the top features ranked by their mean absolute SHAP values **(A)**; SHAP beeswarm plot illustrating the distribution of SHAP values for key functional connectivity features **(B)**.

**Figure 3B** illustrates these effects through a beeswarm plot, where each point represents a participant’s SHAP value for a given feature. The horizontal axis represents the SHAP value (positive values increase the predicted probability of MDD, whereas negative values decrease it), while color encodes the corresponding feature value. Higher FC values (yellow) shifting predictions toward the MDD class, and lower FC values (purple) shifting predictions toward the HC class.

## 4 Discussion

In this study, we developed and internally validated an fNIRS-based ML framework that integrates resting-state and VFT recordings to distinguish MDD from HCs. Using nested stratified cross-validation with OOF evaluation, the SVM achieved good discrimination. SHAP analysis further revealed that FC features contributed more strongly than HbO amplitude measures, suggesting that connectivity-derived signatures carried the most discriminative information in this dataset.

### 4.1 Clinical relevance of an interpretable fNIRS–machine learning framework

The findings support the feasibility of combining fNIRS with ML as an objective approach for MDD-related classification. This is clinically relevant because accessible biomarkers that complement symptom-based assessment remain limited in routine psychiatric practice. In addition, the interpretability provided by SHAP helps address concerns about “black-box” predictions and clarifies the neurophysiological patterns driving the model’s decisions (10).

Our results are broadly consistent with previous fNIRS studies in depression. Previous studies have shown that MDD is associated with attenuated prefrontal HbO responses during tasks such as the VFT, and a meta-analysis has further confirmed reduced PFC HbO responses in individuals with MDD (15). In addition, resting-state whole-head NIRS studies have demonstrated reduced connectivity within cognitive control circuits involving the DLPFC and parietal regions, together with altered prefrontal network interactions in individuals with MDD (4).

At the same time, resting-state fNIRS captures intrinsic network organization and may reveal abnormalities not fully expressed during task-evoked activation. The dual-paradigm design enabled resting-state and VFT-derived information to be evaluated within the same analytical framework. Consistent with this design, highly ranked SHAP features were identified from both resting-state and VFT recordings.

Recent fNIRS studies employing more advanced algorithms have reported comparable or better performance. For instance, Ho et al. used a deep learning model on a large sample (n > 500) and achieved 81% accuracy in distinguishing MDD from HCs (12).

Notably, their interpretable neural network also identified dysregulated frontal regions as key features, which is in agreement with the regions highlighted by our SVM. Another study by Huang et al. focused on differentiating depression severity with fNIRS VFT data, and their SVM classifier reached an accuracy of 92.8% (10). These studies demonstrate the progress achieved in previous fNIRS-based ML classification research. Our study adds to this literature by evaluating FC and regional HbO amplitude features derived from resting-state and VFT recordings within a unified validation and interpretation framework.

### 4.2 Network-level connectivity alterations as key discriminative features

A key finding of our study is that FC features were more informative for MDD classification than regional HbO amplitude measures, with age also contributing but remaining less influential than FC as a group. In the SHAP analysis, connections involving prefrontal regions—particularly links among IPFC, DLPFC, and the TPC—consistently ranked among the most influential predictors. This pattern suggests that MDD in our cohort is not captured solely by localized prefrontal hypoactivation, but also by altered inter-regional coordination between prefrontal and temporal systems. The DLPFC is a core hub for executive function and cognitive control, domains that are commonly impaired in MDD (18). Our results imply that the DLPFC’s interactions with other regions are significantly altered in depression. Accordingly, the prominence of DLPFC-related connectivity in our model may reflect disrupted recruitment or integration of cognitive control circuitry during both resting-state and task-evoked processing.

This interpretation is broadly consistent with prior evidence indicating aberrant FC in depression. For example, Yang et al. reported task-dependent connectivity asymmetries in MDD, with connectivity patterns differing across hemispheres and tasks, alongside atypical lateralization of frontal activation (19). Together, these observations support the view that network-level abnormalities may provide diagnostically relevant information beyond regional activation magnitude alone.

Notably, our results further highlight the importance of the DLPFC and adjacent inferior frontal regions in MDD. These areas are frequently implicated in the neurobiology of depression and are also targeted by therapeutic interventions such as repetitive TMS (20). Our SHAP-based feature importance is broadly consistent with Chao et al., who reported abnormalities in oxygenation within bilateral DLPFC/PFC regions in MDD, together with widespread alterations in inter-channel correlations indicative of network-level dysfunction (21).

In line with this evidence, our findings emphasize that connectivity patterns involving the DLPFC carry substantial discriminative information. One plausible interpretation is that altered DLPFC–TPC coupling reflects impaired coordination between executive control circuitry and temporal regions engaged in semantic or affective processing during tasks such as VFT. However, because fNIRS spatial resolution and probe placement constrain anatomical specificity, the neurocognitive interpretation should be made cautiously and verified in future studies with broader cortical coverage or multimodal imaging. Nevertheless, the convergence of our connectivity-dominant SHAP signature with prior fNIRS reports supports the relevance of prefrontal network dysregulation in MDD.

In sum, the neurophysiological picture emerging from our study supports a network-level account of MDD. Specifically, depression in our cohort was associated with altered synchronization and coordination among prefrontal regions and temporally related channels measured by fNIRS, consistent with disrupted integration of cognitive and affective processing. It is encouraging that SHAP highlighted a set of coherent and interpretable connectivity patterns, which aligns with contemporary circuit-based models of depression and converges with prior fNIRS reports of abnormal prefrontal network connectivity in MDD.

### 4.3 Clinical implications and future translation

Developing an accurate and interpretable brain-based classifier for MDD may have meaningful clinical implications. In principle, an fNIRS-based system that detects depression-related cortical patterns could complement routine assessments by providing an objective and portable neurophysiological signal. First, such a tool could support earlier identification and triage. Because depressive symptoms are often underreported and may remain unrecognized in settings such as primary care or among college students (22), a brief fNIRS protocol could help flag individuals who may benefit from further psychiatric evaluation. In this context, the relatively high sensitivity observed in our model suggests a potential advantage for reducing false negatives, which is important for minimizing missed cases.

Previous resting-state fMRI studies have consistently reported altered FC in MDD, particularly involving default-mode and frontoparietal systems, supporting network-level dysregulation as a core feature of depression (23). However, the cost, limited accessibility, and operational complexity of MRI constrain its feasibility for routine screening or large-scale community deployment. In contrast, fNIRS is non-invasive, portable, and relatively economical, making it a practical modality for capturing cortical hemodynamic correlates of depression-related circuitry in outpatient or community settings. From a translational perspective, our combined resting-state and VFT protocol can be implemented within a relatively short assessment session, and the classifier can output an individualized probability score that may assist clinical triage. The high sensitivity observed in our model suggests potential usefulness for screening applications where minimizing missed cases is prioritized, while model interpretability facilitates transparent communication of the underlying neurophysiological contributors. Nevertheless, given the moderate specificity and the absence of external validation, such a system should be viewed as an adjunct to—rather than a replacement for—standard diagnostic evaluation. With further multicenter validation and prospective testing, interpretable fNIRS-based classifiers may help improve early identification pathways and provide insight into individual variability in depression-related network dysfunction.

Second, the interpretability of the classifier may increase clinical trust and usability. Rather than functioning as a “black-box”, the model can provide feature-level explanations—indicating, for example, that a given prediction is driven by altered prefrontal–temporal connectivity involving DLPFC-related links captured by our fNIRS channel layout. Such explanations can facilitate communication with clinicians and patients and may help generate testable hypotheses about circuit-level dysfunction. Although treatment personalization remains a future goal, interpretable models represent a practical step toward bridging statistical prediction and clinical decision support, provided that external validation and prospective evaluation are achieved.

### 4.4 Limitations and future directions

Despite the encouraging findings, several limitations should be acknowledged. First, participants were recruited from a single site with a moderate sample size, which may limit generalizability across populations, clinical settings, and fNIRS hardware configurations. Although we used nested cross-validation with OOF evaluation to provide a more conservative internal estimate of performance, larger and more diverse multisite cohorts are needed to confirm robustness. In addition, the MDD and HC groups were unequal in size. Stratification in both cross-validation loops maintained approximately consistent class proportions across folds, and threshold-dependent metrics were calculated using the Youden-optimal threshold derived from the OOF ROC curve. The smaller HC group may still limit the precision of class-specific performance estimates. Future studies should validate the model in larger and more balanced multicenter cohorts.

Second, we did not conduct external validation on an independent cohort. Internal validation, even when implemented rigorously, cannot fully substitute for testing on unseen datasets collected at different sites or time periods. A critical next step is to evaluate the classifier on independent external datasets and, ideally, in prospective clinical studies that reflect real-world deployment scenarios.

Third, our fNIRS montage covered only the prefrontal cortex, limiting our ability to characterize large-scale network interactions involving regions such as the parietal or temporal lobes. As a result, the FC features identified by our model should be interpreted as frontal and fronto–temporal patterns rather than whole-brain connectivity signatures. Future studies using wider or whole-head optode coverage will be necessary to determine whether incorporating additional cortical regions improves model performance or yields a more comprehensive understanding of network-level dysfunction in MDD.

Fourth, our feature set primarily focused on HbO-derived measures. Incorporating HbR may provide complementary physiological information regarding oxygen delivery and extraction, and could improve robustness in certain individuals (21). In the present study, regional activation was operationalized using relatively simple mean-level and contrast measures, without incorporating richer hemodynamic response (e.g., slopes). Therefore, the advantage of FC features observed here should be understood within the context of this specific feature space. Likewise, our connectivity metrics were limited to linear correlations; non-linear connectivity, network topology measures or frequency-domain features might further enrich the model. Fifth, the potential influence of psychotropic medications should be considered. Medications commonly used in MDD, including benzodiazepines and antipsychotic agents, may affect cerebral hemodynamic responses and cognitive performance during fNIRS tasks. Because detailed medication profiles were not systematically collected in the present study, we could not determine whether medication status contributed to the observed classification patterns. Future studies should incorporate comprehensive medication assessments and consider medication-related effects when developing fNIRS-based biomarkers for MDD (24). Finally, while SVM provided a good balance between performance and interpretability in our work, it is only one of many ML approaches. We also explored PLS, LDA, and others; their lower performance indicates the importance of non-linear decision boundaries for this dataset. Recent developments have shown even higher accuracies for depression classification (25), though these often lack transparency. In summary, while our study provides proof-of-concept for a fNIRS-based MDD classifier, addressing these limitations – through larger, multi-center validations, expanded feature sets including HbR, and continued refinement of interpretable ML techniques – will be essential before such a tool can be translated into routine clinical use. Our findings lay a foundation for this next phase of research, and we remain optimistic that with these improvements, fNIRS could become a valuable component of the clinical toolkit for depression diagnosis and management.

## 5 Conclusions

This study demonstrates that integrating resting-state and VFT-based fNIRS features with ML can effectively distinguish individuals with MDD from healthy controls. Among the evaluated classifiers, the SVM model achieved the best overall performance, and SHAP analysis further revealed that functional connectivity features provided greater discriminative information than regional HbO amplitude measures, highlighting the importance of network-level dysfunction in MDD-related neurophysiological alterations.

The findings suggest that an interpretable fNIRS-based ML framework may provide a potential adjunctive approach for objective assessment of MDD. However, the current results should be considered preliminary because they were obtained from a single-site cohort without external validation. Future studies should focus on larger and more balanced multicenter cohorts, standardized fNIRS acquisition and preprocessing protocols, external validation across independent datasets, and incorporation of additional physiological features such as HbR and advanced connectivity metrics. Longitudinal studies are also warranted to determine whether fNIRS-based models can capture treatment-related changes and contribute to personalized clinical decision-making.

## Data Availability

The raw data supporting the conclusions of this article will be made available by the authors, without undue reservation.
